# Lumbar spine abnormalities and facet joint angles in asymptomatic elite junior tennis players

**DOI:** 10.1186/s40798-020-00285-4

**Published:** 2020-11-25

**Authors:** Molly Connolly, Andrew H. Rotstein, Justin Roebert, Rafal Grabinski, Frank Malara, Tomas O’Shea, Tim Wood, Melanie Omizzolo, Stephanie Kovalchik, Machar Reid

**Affiliations:** 1 Institute for Health and Sport, Victoria University, Melbourne, Australia; 2 Performance, Tennis Australia, Melbourne, Australia; 3 Victoria House Medical Imaging, 435 Malvern Rd, South Yarra, Melbourne, Australia; 4 Glenferrie Private Hospital, Melbourne, Australia; 5 Game Insight Group, Tennis Australia, Melbourne, Australia

**Keywords:** MRI, Lumbar spine, Tennis, Injury, Facet joint

## Abstract

**Background:**

Lumbar spine abnormalities, in particular stress fractures to the pars interarticularis, are common in elite junior tennis players, though the difference in prevalence between males and females remains unclear. Further, facet joint orientation appears to be a possible option for recognizing which players might go on to present with a pars stress fracture. Given the link between pars stress fractures and low back pain in tennis players, it appears logical to explore the link between facet joint angle and pars abnormalities. Thus, the purpose of this study was to describe the prevalence of lumbar spine abnormalities and explore the relationship between facet joint orientation and pars abnormalities in elite adolescent tennis players.

**Methodology:**

Lumbar spine MRI images of 25 elite junior tennis players were obtained and distributed between five radiologists for analysis. Descriptive comparisons and confidence intervals were used to describe the prevalence of the abnormalities. A generalized linear regression model was conducted to investigate the relationship between lumbar pars abnormalities and lumbar facet joint angles.

**Results:**

Sixteen (64%) of 25 players were found to have at least one lumbar spine abnormality. Pars abnormalities affected 36% of players while bone marrow edema was found in 24% of players. Disc herniation, disc degeneration, and facet joint degeneration were diagnosed in 20%, 44%, and 24% of players respectively. Lastly, one player (4%) was diagnosed with spondylolisthesis. Females had significantly larger facet joint angles across L3/4 L5/S1 compared to males (*p* < 0.01). Further, those who had pars abnormalities had larger facet joint angles compared to those who did not (*p* < 0.001).

**Conclusion:**

Disc degeneration, pars abnormalities, including bone marrow edema, and facet joint degeneration were common findings among elite adolescent tennis players.

Additionally, this study is the first to discover that pars abnormalities are linked to facet joint angle in elite adolescent tennis players. This finding might assist in identifying tennis players at a greater risk of developing lumbar spine pars abnormalities in the future.

## Key points

- Asymptomatic lumbar spine abnormalities were present in 64% of elite adolescent tennis players in this study.
- Disc degeneration, pars interarticularis abnormalities (including bone marrow edema), and facet joint degeneration were common findings among elite adolescent tennis players.
- Lumbar spine pars abnormalities were found to be linked to facet joint orientation. This finding might help practitioners to identify players at risk of developing a pars abnormality.

## Introduction

Elite junior tennis players commonly sustain lumbar injuries. Unpublished data from Tennis Australia states that between 2008 and 2016 approximately 60% of players aged 11–19 years who presented with low back pain (LBP) were diagnosed with a symptomatic pars abnormality (bone marrow edema (BMO) with or without a fracture evident). Further, athletes were reported to take ~ 160 days before returning to play following these diagnoses.

Given that this interruption to tennis play intersects with a critical stage of the athletes’ development [1], the importance of preventing these injuries cannot be understated. Additionally, the vast majority of injured players were male, inferring an apparent gender-based discrepancy.

Low back pain (LBP) is a symptom that arises due to either acute or repetitive micro-trauma, or can be due to a combination of both in young athletes [2]. Despite the insidious nature of low back pain in tennis players, empirical evidence linking risk factors with causation are limited. For example, prior research has found that pars abnormalities as well as facet joint arthropathy, disc degeneration, and disc herniation are among the most common abnormalities in asymptomatic adolescent tennis players [3–5]. However, there has been no longitudinal research linking these abnormalities to LBP or gender in tennis.

Morphological components of the vertebrae have also been explored as a potential risk factor in LBP [6–12]. Facet joints with a more coronal appearance and larger facet joint angle have been associated with pars abnormalities [8, 12]. Intuitively, there seems scope to consider the possibility that coronally oriented facet joints will strongly relate to pars abnormalities in younger athletic populations. In addition, previous research has found that the tennis serve, especially the kick serve [13], induces higher loading in the spine compared to groundstrokes [14]. Typically, the kick serve is characterized by a more lateral racquet/ball impact position compared to the flat serve [15] which requires more spinal lateral flexion in order to make contact with the ball when serving [5]. Further, left lateral flexion forces have been linked to LBP in elite adolescent tennis players with players experiencing lumbar loading up to four times their body weight during serving [16]. Significant lumbar loading, when coupled with more coronally facing facet joints, may explain the high number of pars abnormalities in young tennis players, though this hypothesis remains untested.

To the knowledge of the authors, no previous study has compared the lumbar abnormalities of asymptomatic male and female tennis players at a key risk age, between 11 and 16 years old. Therefore, the purpose of this descriptive study was to describe the prevalence of lumbar spine abnormalities and explore the relationship between facet joint orientation and pars abnormalities in elite adolescent male and female tennis players. The findings of this study will serve as baseline information for a prospective study investigating lumbar pain and subsequent lumbar injuries in elite adolescent tennis players. We hypothesize that a large majority of players will be diagnosed with an asymptomatic lumbar abnormality but that males will be diagnosed with more abnormalities, specifically pars abnormalities, than females.

## Materials and methods

### Participants

Magnetic resonance imaging (MRI) scans of the lumbar spines of twenty-five (male 14, female 11) right-handed elite adolescent tennis players aged 13 ± 1.7 years (range 11–17 years), who were part of a National Tennis Academy, were obtained as part of an annual screening protocol (between March and May 2017). All participants were free of low back pain, any current performance inhibiting injury or illness at the time of scanning and were excluded if they had reported 7 or more consecutive days of LBP during the last 6 months or had experienced LBP with an accompanying positive MRI (which then resulted in modified workload). All players were right-handed which means that abnormalities described as right sided will be synonymous with the dominant side.

Ethical approval was obtained from the Victoria University Human Research Ethics Committee while participants provided voluntary informed consent and assent prior to any involvement in the study. This study was performed in accordance with the standards of ethics outline in the Declaration of Helsinki.

### Imaging technique

All magnetic resonance imaging (MRI) was carried out using 3-T Siemens Verio and Vida scanners, Erlangen Germany. The following standard sequences were performed. Sagittal T2, TR 4880 ms, TE 43 ms, FOV 260 mm, Matrix 384 × 384, slice thickness 3.5 mm, 4.2 mm separation. Sagittal STIR, TR 4020 ms, TE 53 ms, FOV 300 mm, Matrix 384 × 384, slice thickness 3 mm, separation 3.75 mm. Sagittal T1, TR 550 ms, TE 11 ms, FOV 260 mm, Matrix 768 × 768, slice thickness 3.5 mm, separation 4.2 mm. Axial T2 TR 3380 ms, TE 87 ms, FOV 240 × 240 mm, Matrix 448 × 444, slice thickness 4 mm, separation 4.4.mm. Sagittal T1 3D fat-saturated VIBE, TR 7 ms, TE 2.5 ms, FOV 200 × 200 mm, Matrix 256 × 256, slice thickness 2 mm. Parasagittal T1 fat-saturated VIBE images were reformatted through the lumbar pars interarticularis at 1-mm thickness.

### Data collation

To ensure the abnormality gradings were consistent between the radiologists, an inter-rater reliability was established.

Five MRI scans which included abnormalities of interest were sourced externally and provided to the radiologists to become familiar with the grading systems provided by the lead researcher. These scans were de-identified scans of other patients of the clinic that had undergone a lumbar spine scan. The scans chosen included abnormalities that the radiologists were required to grade in the study. Five experienced musculoskeletal radiologists assessed these five scans for familiarization of the abnormalities that would be assessed for the project. The five scans were assessed for the presence and severity of pars abnormalities, BMO, disc herniation, nerve root compression, canal stenosis, foraminal stenosis, disc degeneration, annular fissure, Modic changes, Schmorl’s nodes, Scheuermann’s disease, facet joint orientation, facet joint degeneration, facet synovial cysts, spondylolisthesis, and spina bifida occulta. Each abnormality was graded using a peer-reviewed grading system within the literature unless deemed irrelevant (whereby yes/no was used to indicate presence of an abnormality) (Table 1). Once the radiologists had discussed and mutually agreed upon the grading systems and the specific grades corresponding to the five scans they reviewed together [28], they then completed a reliability study.

**Table 1:** List of abnormalities and the corresponding grading systems used in this study

Abnormality	Grading System used	Classifications
Pars abnormality	Ang et al. [17]	Grade 0 = normal, grade 1 = a stress reaction, grade 2a = an active incomplete fracture, grade 2b = a chronic incomplete fracture, grade 3 = active complete fracture, grade 4 = chronic complete fracture
Bone marrow edema	Sims et al. [18]	The severity is calculated from the sagittal STIR sequence. The intensity value of bone marrow at the site of edema is measured using the region of interest tool. This value is then divided by the value of normal bone marrow within the vertebral body at the same level of the pars. This is referred to as the BMO “ratio”
Disc herniation	Mysliwiec et al. [19]	Grading is dependent upon the direction and the magnitude of the disc protrusion. The grading depends on the magnitude of the distance the protrusion extends posteriorly (grading is between 1 and 3), and then follows with the mediolateral location of the protrusion (grade A–C).
Nerve root compression	Pfirrmann et al. [20]	Grade 0 = normal, grade 1 = contact, grade 2 = deviation, or grade 3 = compression
Canal stenosis	Guen et al. [21]	Grade 0 = normal, grade 1 = mild, grade 2 = moderate, grade 3 = severe
Foraminal stenosis	Park et al. [22]	Grade 0 = normal, grade 1 = mild, grade 2 = moderate, grade 3 = severe
Disc degeneration	Pfirrmann et al. [23]	Grade 0 = normal through to grade 5 = severe
Annular fissure	Yes/no answer with location (right, posterior/central or left)	“Yes” or “No”
Modic endplate changes	Modic et al. [24]	“Type 1” (decreased signal intensity on T1-weight images, increased signal intensity in T2-weighted images), “Type 2” (increased signal on T1-weighted images and a slightly hyperintense signal on T2-weighted images), or “Type 3” (decreased signal intensity on both T1- and T2-weighted images)
Schmorl’s nodes	Yes/no answer	“Yes” or “No”
Scheuermann’s disease	Yes/no answer	“Yes” or “No”
Facet joint orientation	Noren et al. [25]	(Adapted method from Noren et al.) Facet joint orientation was measured using a midsagittal line through the vertebral body and the intersecting lines passing over the endpoints of each facet (see Fig. 1).
Facet joint degeneration	Weishaupt et al. [26]	Grade 0 = normal through to grade 3 = severe
Facet synovial cyst	Yes/no answer with location (left/right and infraspinal/extraspinal)	“Yes” or “No” with “Right” or “Left”
Spondylolisthesis	Meyerding [27]	Grade 0 = no spondylolisthesis, grade 1 = 1–25% vertebral slip, grade 2 = 26–50% vertebral slip, grade 3 = 51–75% vertebral slip, grade 4 = 76–100% vertebral slip, or grade 5 = > 100% vertebral slip
Spina bifida occulta	Yes/no answer	“Yes” or “No”

Thirty de-identified lumbar spine scans were sought by a clinician external to the study (working at the clinic where the radiologists are based) that included specific lumbar spine abnormalities (pars abnormalities, BMO, and disc herniation) to use for the reliability study. Each radiologist was asked to grade pars abnormalities, BMO, disc herniation, and facet joint orientation for every scan (all radiologists assessed the 30 scans). These abnormalities were chosen for the reliability study as they are believed to be the most difficult (or potentially variable) to grade due to the large number of grading categories and attention to detail required to correctly diagnose each abnormality. The remaining abnormalities (Table 1) were deemed straight forward to grade and identify and therefore were determined unnecessary for the reliability study.

Agreement on ratings was measured with Fleiss’s kappa for abnormalities with a categorical grading and with the intra-class correlation (ICC) for abnormalities graded on a continuous scale. Excellent agreement was found in the reliability results for pars abnormalities (ICC 0.95), BMO (ICC 0.93), and facet joint angles (ICC 0.86). For disc herniation, where grading was on a nominal scale, the kappa was 0.51. Lower values for agreement when using the kappa statistic are expected in comparison to the ICC, as kappa ignores any ordinality in ratings and high values of agreement are less likely as the number of rating categories increases [29]. General guidance suggests values between 0.41 and 0.6 as “moderate” reliability for kappa and 0.6 to 0.74 for the ICC [30]. In our study, the observed agreement between raters was 83% [30]. Together, these results suggest that there was good reliability among raters and the most difficult categories of abnormalities.

Once reliability was established, the MRI scans from the sample of elite players (*n* = 25) were randomly distributed between the radiologists (five scans each) by an independent researcher for detailed review. For these scans, the radiologists assessed all lumbar abnormalities featured in Table 1 using the nominated grading system.

Among a number of lumbar spine abnormalities, this study analyzed both pars abnormalities and BMO severity. Pars abnormalities refer to morphological changes to the pars interarticularis as described by Ang et al. [17]. This grading system describes pars abnormalities as follows: normal (grade 0), a stress reaction (grade 1), an active incomplete fracture (grade 2a), a chronic incomplete fracture (grade 2b), an active complete fracture (grade 3), or a chronic complete fracture (grade 4). Three of these grades (grades 1, 2a, and 3) include BMO; however, the severity of the BMO is not described. Therefore, we have also included a BMO grading system by Sims et al. [18] in this study to describe the severity of BMO found on the MRI images. The severity of BMO is calculated from the sagittal STIR sequence. The intensity value of bone marrow at the site of edema is measured using the region of interest tool. This value is then divided by the value of normal bone marrow within the vertebral body at the same level of the pars. This product value is referred to as the BMO “ratio”. Sims et al. [18] state that a ratio close to 2 reflects early stage asymptomatic lumbar bone stress. A ratio between 2 and 3 signifies clinically significant BMO which is likely to be symptomatic, and a ratio of 3 or more is likely to represent the later stages of symptomatic lumbar bone stress injury.

### Statistical analysis

Due to being a descriptive study, descriptive comparisons are mostly presented in the results and discussion. Age characteristics of the sample were summarized with the mean and standard deviation. The incidence of abnormalities was summarised by the percentage occurrence and 95% confidence intervals. Qualitative comparison of gender differences was also performed where appropriate. A generalized linear regression model was conducted to determine the relationship between (1) those with pars abnormalities and facet joint orientation angle and (2) between sex and facet joint orientation angle. Values of *p* < 0.05 were considered statistically significant. All analysis was performed with the RStudio software (version: 0.99.903, RStudio: integrated Development for R. RStudio, inc., Boston, MA).

## Results

Sixteen out of 25 players (64%, 95% CI 43 to 81%) (10 male, 6 female) were found to have at least one abnormality (Table 2). All players were right-handed which means that abnormalities described as being on the dominant side will be synonymous with their right side, and abnormalities described as being on the non-dominant side will be synonymous with their left side.

**Table 2:**
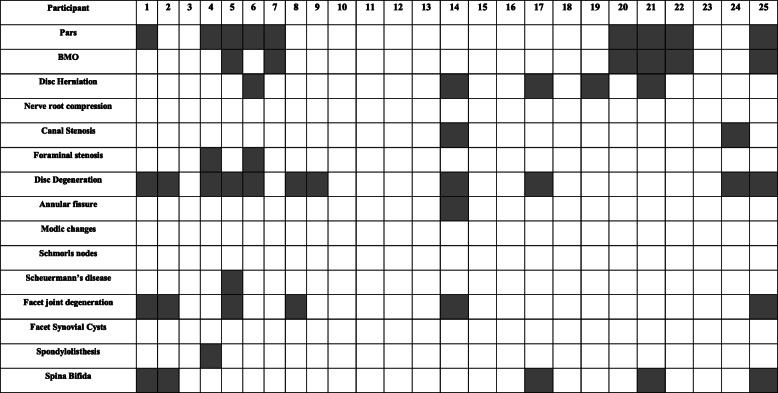
A list of the participants and their respective lumbar spine abnormalities detected

Grey = diagnosed, white = not diagnosed

### Pars abnormalities

A total of 9 out of 25 (36%, 95% CI 19 to 57%) players had pars abnormalities (7 males, 2 females), at L4 and L5. None was present between L1 and L3. Two players had abnormalities at L4, and seven players had abnormalities at L5. Three players had isolated right side pars abnormalities while the remaining six players had bilateral pars abnormalities. No players had isolated left side pars abnormalities. Seven instances of grade 1 (a stress reaction) pars abnormalities were detected, two instances of grade 2a (an active incomplete fracture), one instance of grade 2b (a chronic incomplete fracture), and five instances of grade 4 pars abnormalities (chronic complete fracture). No instances of grade 3 pars abnormalities (active complete fracture) were detected in this study.

### BMO

Six out of 25 players had BMO (5 males, 1 female) (24%, 95% CI 10 to 46%), all at L4 or L5. Two male players had BMO at L4, one of which had BMO present on the dominant (right) side at the pedicle (posterior side) and the pars while the other had BMO on the non-dominant (left) side at the pedicle (posterior and anterior sides) as well as at the pars. The player with BMO on the dominant side had a BMO ratio above 2; however, the player with BMO on the non-dominant side had a BMO ratio of less than 2 (Table 3). Three males had BMO (1 male: dominant side, 2 males: bilateral) at L5 at the pars, the pedicle (posterior side), and extending into the vertebral body. Two of the three males also had BMO (bilateral) at the pedicle on the anterior side, transverse process, and the superior articular process while the other male had BMO (dominant side) at the inferior articular process. Of the male players with bilateral L5 BMO, one had a BMO ratio exceeding 3 on both sides; the other male had a ratio exceeding 2 on the dominant side and a BMO ratio less than 2 on the non-dominant side. The male who had BMO at L5 on the dominant side had a BMO ratio exceeding 2. One female had bilateral BMO at L5 which was only present at the pars and had a BMO ratio of less than 2.

**Table 3:** A list of the participants with BMO and their respective BMO details

Participant	Gender	Ratio right pars	Ratio left pars	Diagnosis	Level
1	Male	4.4	3.6	Bilateral	L5
2	Male	2.1	1.1	Right	L4
3	Male	2.6	0.9	Right	L5
4	Male	2.2	1.5	Bilateral	L5
5	Male	1.2	1.7	Left	L4
6	Female	1.7	1.8	Bilateral	L5

### Disc abnormalities

#### Disc herniation

Five of the 25 players (4 male, 1 female) (20%, 95% CI 8 to 41%) had disc herniation either at L4/5 or L5/S1. One female and one male had disc herniation at L4/5 with gradings of 1A and 1C respectively. Three males demonstrated disc herniation at L5/S1 with two gradings of 1A and one grading of 1C. All abnormalities demonstrated a small bulge into the lumbar canal with three bulges being central and one being lateral.

#### Nerve root compression

No instances of nerve root compression were found in this cohort.

#### Canal and Foraminal stenosis

Two of the 25 players (male 0, females 2) (8%, 95% CI 1 to 28%) had canal stenosis. One player had a grade 1 canal stenosis at L4/5, and the other player had grade 1 stenosis at both L4/5 and L5/S1. Two out of 25 players had foraminal stenosis (male 1, female 1) (8%, 95% CI 1 to 28%), both at the L5/S1 level. The male and female player had a grade 1 and a grade 3 foraminal stenosis respectively.

#### Disc degeneration

Eleven out of the 25 players (5 males, 6 females) (44%, 95% CI 25 to 65%) had some degree of disc degeneration within the lumbar spine with 30 instances of grade 2 disc degeneration. Five, five, and seven instances of grade 2 degeneration were found at L1/2 (two males and 3 females), at L2/3 (2 males and 3 females), and at level L3/4 (4 males and 3 females) respectively. At level L4/5, 6 instances of grade 2 degeneration were detected in 4 males and 2 females. Lastly, 4 males and 3 females were detected with grade 2 disc degeneration at L5/S1. While there were some players who had multi-level disc degeneration (7 players: 4 males, 3 females), there were 4 players (1 male, 3 females) who had single level of disc degeneration.

#### Annular fissures

One player had a posterior/central annular fissure (male 0, female 1) (4%, 95% CI 0 to 22%) at level L4/5.

### Endplates

No Modic changes or Schmorl’s nodes were found within this cohort.

#### Scheuermann’s disease

Only one male was found to have Scheuermann’s disease.

### Facets

#### Facet degeneration

Six of the 25 players (3 males, 3 females) (24%, 95% CI 10 to 46%) had grade 1 facet joint degeneration with a total of 16/125 (12.8%) facet joints affected. No degeneration was detected at the L1/2 or L2/3 joints in male or females. Five instances of facet degeneration occurred at L3/4 across 3 players. One male and one female had bilateral facet degeneration while there was one case of right facet joint degeneration in a female player. Seven instances of facet degeneration were found at L4/5 in 4 players (1 male, 3 females), with three cases of bilateral degeneration and one case left facet joint degeneration in a female player. Lastly, five instances of facet degeneration were found at L5/S1 in 3 players (2 males, 1 female). A single male and female had bilateral facet degeneration while the other male had degeneration of the right facet joint.

#### Facet orientation

**Fig. 1:**
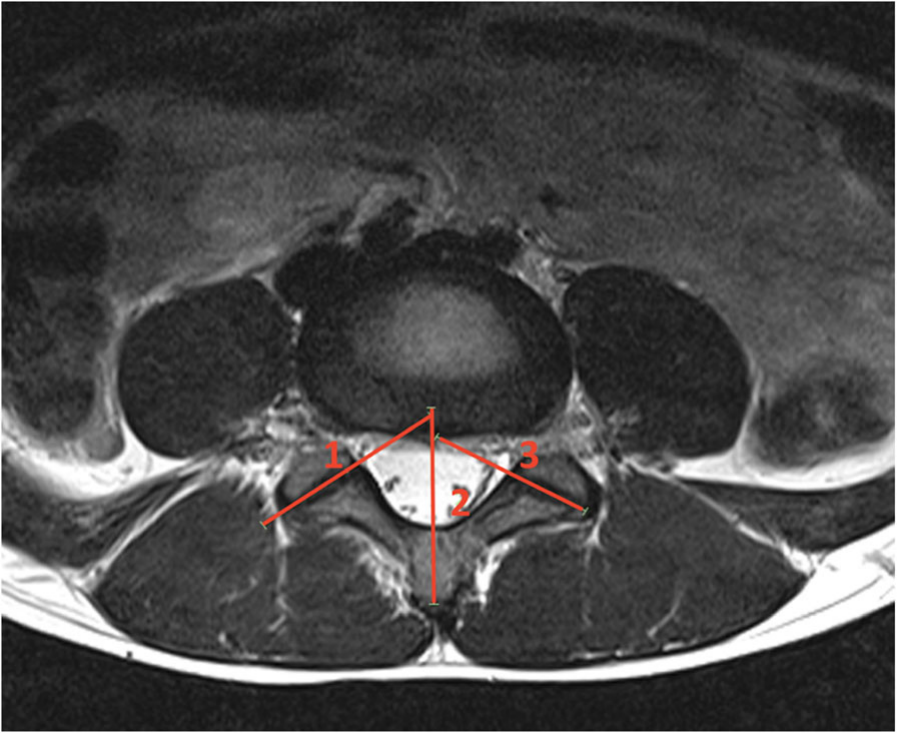
Facet joint angle measurement. Lines 1 and 3 pass through the facet joints being measured

One hundred and fifty facet joint angles were calculated for this study using methods established by Noren et al. [25] (Fig. 1). The average facet joint angles at each level are presented in Table 4.

**Table 4:** Mean facet joint angles measured using grading system used in Noren et al. [25]

	Male		Female	
Facet joint	Pars	No pars	Pars	No pars
Right facet angle—L3/4	41.9	37.4	40.9	41.5
Left facet angle—L3/4	42.9	35.5	49.7	41.9
Right facet angle—L4/5	53.6	44	54.3	51
Left facet angle—L4/5	55.5	47.2	64.2	52.3
Right facet angle—L5/S1	47.2	45.6	51.5	51.2
Left facet angle—L5/S1	51.2	48.6	75.2	51.8

Results from the generalized linear regression model found that those with pars abnormalities had significantly larger facet joint angles compared to those without pars abnormalities (*p* < 0.001). Further, females had significantly larger facet joint angles compared to males (*p* < 0.01).

#### Synovial cyst

No synovial cysts were found in this cohort.

#### Spondylolisthesis

One female player had spondylolisthesis (4%, 95% CI 0 to 22%) of grade 1 severity.

#### Spina bifida

Five of the 25 players (4 male, 1 female) (20%, 95% CI 8 to 41%) had spina bifida. All instances were at S1 with no other level affected.

## Discussion

Adolescent tennis players commonly demonstrate asymptomatic lumbar abnormalities on MRI, though there is little research available about the nature of these abnormalities and their relationship with future LBP. Adolescent tennis players commonly sustain LBP [3, 4]; however, junior male players have been found to be more susceptible to LBP than female players according to unpublished data from Tennis Australia. While it is well established that asymptomatic lumbar spine abnormalities are prevalent in young tennis players, the link between such abnormalities and the risk of developing LBP is currently unclear. Also as a result, it would be wise to exercise caution when interpreting lumbar spine MRI results in players with LBP. The discussion below will critique the results with clinical and practical implications detailed.

### Pars abnormalities

Abnormalities of the pars interarticularis are known to be the greatest cause for LBP within the Tennis Australia National Academy. Within this study, pars abnormalities were the second most common abnormality found in this sample, affecting 36% of players. As hypothesized, the prevalence of pars abnormalities was lower in female players than in male players (18.2 vs 50% respectively) although the overall incidence rate of pars abnormalities in our sample was slightly higher than previous studies. Alyas et al. [3] reported 9 pars abnormalities in their sample of 33 (mean age 17 ± 1.7 years, 18 junior males, and 15 females) players (27% incidence). Our study’s higher incidence rate may relate to the younger age of our cohort (mean age 13 ± 1.7 years) with the ossification of the neural arch not yet complete [31].

Interestingly, the majority of the pars abnormalities were bilateral, with a few on the dominant side only. This suggests that perhaps the preferred hitting arm does not have a significant impact on the injury site in tennis players whereby for comparison, cricket players predominantly suffer pars injuries on the side contralateral (or non-dominant) to the bowling arm [32, 33]. Previous reports have shown that adolescent cricket bowlers demonstrate around 27.5° of left lateral flexion and almost 44° of flexion during the delivery stride [34]. These peak findings are approximately 17.5° and 38° greater than the lumbar left lateral flexion and lumbar flexion (respectively), reported in tennis players when serving [5]. Another report suggested that the combination of lateral flexion and axial rotation significantly increases the load in the facet joints [35]. With this in mind, Burnett et al. [34] reported that peak lateral flexion on the non-dominant side (left) was greater compared to the dominant side (right) (left 27.5°, right 4.4°), which in combination with the axial rotation involved in fast bowling might explain why fast bowlers ted to experience lumbar injuries on the non-dominant side. On the contrary, tennis players exhibit much less flexion (approximately − 6–6° when serving) though have much greater extension (up to 22°) compared to bowlers [5]. Without one side experiencing much greater lateral flexion (like in cricket), it might be that lumbar extension plays a more pivotal role in the onset of lumbar injuries in tennis players.

As alluded to above, seven male players had pars abnormalities as compared to two females. Grade 1 followed by grade 4 was the most frequent type of pars abnormalities among the males while the two females had an even distribution of grades 1 and 4 abnormalities. Interestingly, the affected males tended to be older (12–16 years of age) than the females (11–12 years). The introduction of the kick serve into regular practice for these adolescents, which is more commonly practiced and performed in male athletes, stands out as a possible explanation for the disparate incidence between genders. That is, the repetitive hyperextension involved in the kick serve has been speculated as a potential cause for pars injuries [36], particularly when combined with rotation and side flexion [35]. The pars interarticularis provides conduit between the superior and inferior facet joints and as a result is simultaneously sheared, stretched, and subjected to large loads during extension when serving [13, 37].

Those who had grade 1 or grade 2a pars abnormalities had BMO (6 players: 5 males, 1 female). Four of the six players had at least one side (dominant or non-dominant) with a BMO ratio of 2 or more (Table 3), indicating that these players have clinically relevant BMO and should be monitored [18]. The remaining two players (players 5 and 6 in Table 3) had BMO values of less than 2 and thus have a “normal” amount of BMO according to Sims et al. [18]. Arguably though, these players should still be monitored for the following months given some of their signal intensity ratios are closer to a value of 2. The signal intensity ratios are currently used as a guideline to prevent bone stress injuries in elite cricket players. Whether these guidelines are also applicable to junior tennis players will be tested throughout the remainder of this prospective study.

Indeed, those who had BMO were older (aged between 12–16 years), compared to those who did not have BMO but had chronic pars fractures (who were aged 11–13 years). A possible explanation for this could be that the transition from club tennis to elite academy training is characterized by greater volume and intensity of ball striking, which can heighten the stress on the lumbar spine. Academy players are often scouted as young as 10 years of age and recruited around the age of 11 and 12 years. The transition into elite training can also coincide with new coaching, possible strength and conditioning interventions, and changes in equipment. These variables could all contribute as potential risk factors for developing pars abnormalities.

Another potential consideration in the onset of these injuries is puberty. Typically, puberty commences slightly earlier in females than males (9–13 years and 10–14 years respectively) [38]. Given that the pars abnormalities were evident among younger females, the onset of puberty may be linked to a heightened susceptibility of pars abnormality.

Bone mineral density (BMD) and bone growth may also play part in BMO findings. It is known that BMD peaks following peak in height velocity [39]; however, this is not instantaneous. Thus, it is hypothesized that there is a period of time between peak height velocity and BMD when the bone is weaker and vulnerable [40]. Spinal vulnerability following bone growth coupled with increased training loads suggests that junior tennis players could be at a higher risk of low back injuries [41].

### Disc degeneration

Disc degeneration was the most common finding in this study. Disc degeneration was found in 44% of players—slightly higher than another examination of adolescent tennis players (39% prevalence) [3] but lower than a similar study analyzing disc degeneration in elite tennis players with a mean age of 18 years, which reported a prevalence of 62% [4]. The work of Rajeswaran et al. [4] revealed a higher incidence of disc degeneration among males, which contrasts with our study where disc degeneration was more common in female players. Once more, the difference in the age demographic, and therefore physical development stage and training regimen, of the respective cohorts may account for this difference.

### Facets

Facet joint arthropathy was a common finding among this cohort (third most common), affecting 24% of players and approximately 12.8% of all facet joints. Facet joints are a load-bearing conduit between the vertebrae in the spine. Therefore, it was not surprising that facet joint arthropathy was a common finding given the spine is subjected to significant load during the tennis serve [13]. More specifically, during the windup phase of the serve, the lumbar spine is subjected to significant stress due to the center of mass of the upper body sitting behind the body during lumbar extension [42]. Given this extension loading, it follows that the facet joint degeneration was primarily found at L4/5 and L5/S1, similar to previous work [3, 4]. That the degeneration was mostly bilateral was surprising as previous research has found that highly skilled players with greater lateral flexion have greater asymmetric loads on the lumbar spine during the serve, implying that facet joint degeneration may favor one side [42].

At L3/4 and L4/5, the facet orientation was reasonably symmetrical. However, this was not the case at L5/S1, with considerable asymmetry across all players. Interestingly, while there was a natural increase in facet angle from L3/4 to L5/S1, more than half of the facet angles at L4/5 were greater than those at L5/S1. The facet joint angles in this study were consistent with the work of Noren et al. [25] despite study’s participants having a mean age of 32 years and there being some suggestion of facet joint angles decreasing with age [43].

Our study found that those with pars abnormalities had greater facet joint angles compared to those without pars abnormalities. A possible reason for this could be due to the additional strain the pars is under when the facet joints are more frontally oriented as there is greater joint surface area during flexion/extension of the spine [12]. Tennis requires repetitive flexion and extension of the lumbar spine and thus over time could lead to pars abnormalities in tennis players. Furthermore, this could especially affect young tennis players whose spines have not yet fully developed. However, our study also found that females had significantly greater facet joint angles compared to males, although more males had pars abnormalities compared to females (7 males, 2 females). That females have greater facet joint angles and a low incidence of pars abnormalities is intriguing. A possible reason for this contradiction could be that males are simply loading the spine more than female players—especially since junior male tennis players tend to learn and utilize the kick serve (which is known to impart greater lumbar spine loads) more often compared to female players.

Our findings partially support the research of Don and Robertson [8] who, while having a broader range of ages (13–84 years), found that those who had larger facet joint angles had a pars abnormality.

### Disc herniation

This study used an objective measurement system established by Mysliwiec et al. [19] whereby disc herniations were classified using their position relative to other anatomical landmarks. Disc herniation was found in 20% of the players (1 female, 4 males). This incidence is marginally lower than previous tennis studies (Alyas et al. [3] 39% and Rajeswaran et al. [4] 31%) but higher than in asymptomatic non-athletes (16%) [44]. It has been documented that flexion in combination with axial loading places enormous pressure on the annular fibrosis [45] and can contribute to disc herniation.

### Synovial cysts

No synovial cysts were found in this cohort, which contrasts with other studies in males and females [3, 4]. While the etiology of these cysts is unclear [46], it has been reported that there is a strong association between synovial cysts, degenerative spondylolysis, and facet joint arthropathy [47–49], and they are known to manifest where axial rotation demands are high. The absence of cysts among this study’s 25 adolescent players may relate to (a) only mild facet joint degeneration being found and (b) cysts being most common in later stages of life [46].

Our study had a couple of limitations. Firstly, this study had a convenience sample drawing on the limited number of elite adolescent tennis players in the available academy without previous lumbar pathology. This limits the generalizability of the findings. Secondly, we are unable to control for the participants’ current extracurricular activities and their influence on lumbar morphology, notwithstanding that the players’ current playing level was standardized.

## Conclusion

The use of MRI is a valuable tool for determining lumbar spine pathologies that may contribute to the development of low back pain. This study found that disc degeneration, pars abnormalities, including BMO, and facet joint degeneration were common findings among elite adolescent tennis players. Since all players were asymptomatic, this study highlights the need to exercise caution when using MRIs to assist in diagnosing junior tennis players with LBP owing to the abundance of abnormalities that exist asymptomatically.

Males had a higher prevalence of pars abnormalities, BMO, and disc herniation compared to females, although females had a higher prevalence of disc degeneration and facet joint arthropathy. Those players with a pars abnormality had significantly larger facet joint angles than players with a normal pars. This finding could be useful in determining athletes at risk of pars stress fracture injury.

## Data Availability

The dataset for this study will not be publicly available due to an IP agreement between Tennis Australia and Victoria University.

## Abbreviations

LBP: Low back pain
BMO: Bone marrow edema
MRI: Magnetic resonance imaging
